# Laparoscopic artificial insemination in small ruminants: technological integration, economic evaluation, and future perspectives

**DOI:** 10.3389/fvets.2025.1667887

**Published:** 2025-10-10

**Authors:** Ting-Chieh Kang, I-Ling Lai, Perng-Chih Shen

**Affiliations:** 1 Taiwan Livestock Research Institute, Ministry of Agriculture, Pingtung, Taiwan; 2 Graduate Institute of Bioresources, National Pingtung University of Science and Technology, Pingtung, Taiwan; 3 Department of Animal Science, National Pingtung University of Science and Technology, Neipu, Pingtung, Taiwan

**Keywords:** laparoscopic artificial insemination, small ruminants, precision reproductive management, artificial intelligence, computer vision, emerging technologies

## Abstract

Laparoscopic artificial insemination (LAI) has emerged as a cornerstone technology for genetic improvement in small ruminants. By directly depositing semen into the uterine horns through minimally invasive surgery, LAI effectively bypasses the cervical anatomical barrier that hinders transcervical insemination. This approach has elevated pregnancy rates with frozen–thawed semen from 20 to 40% using conventional methods to 60–70%, establishing LAI as the “gold standard” in small ruminant reproduction. This review develops an integrated framework centered on LAI, systematically highlighting how emerging technologies directly enhance its precision and automation. Artificial intelligence–driven multivariate prediction models now enable pregnancy rate forecasting (AUC = 0.86), while computer vision technologies provide highly accurate estrus detection (98.56% accuracy), optimizing insemination timing. Robotic-assisted systems further refine surgical precision, and the integration of Internet of Things (IoT) and digital twin platforms enables end-to-end intelligent reproductive management. Economic evaluations indicate that LAI delivers significant returns on investment when the genetic value of disseminated germplasm is sufficiently high. Although technical complexity and equipment costs remain challenges, the integration of LAI with emerging technologies is driving a paradigm shift toward precision and intelligent management in the small ruminant industry, offering critical support for global food security and sustainable development.

## 1 Introduction

The global small ruminant industry faces the dual challenge of enhancing both production efficiency and genetic quality. With the increasing demand for animal protein driven by population growth and the ongoing threats of climate change, livestock systems must achieve higher productivity under limited resources (1, 2). Genetic improvement remains the most fundamental pathway to enhancing herd performance, and efficient assisted reproductive technologies are critical tools for accelerating the dissemination of superior genes (3). Among these technologies, artificial insemination [Artificial intelligence (AI)] is widely adopted for maximizing the genetic potential of elite sires. However, conventional transcervical artificial insemination [Transcervical artificial insemination (TCAI)] is constrained by the unique anatomical complexity of the sheep and goat cervix, characterized by tortuous spiral folds that create a natural barrier to sperm transport (4, 5). While these structures may have evolved to provide reproductive protection, they represent a major bottleneck in AI. Pregnancy rates achieved with TCAI average only 40–60% when fresh semen is used, and drop further to 20–40% with frozen–thawed semen considerably lower than the 70–80% success rates reported in cattle (5, 6). Laparoscopic artificial insemination (LAI), first introduced in the early 1980s, addressed this limitation by directly depositing semen into the uterine horns through minimally invasive surgery (7–9). Since then, LAI has become the “gold standard” for small ruminant reproduction, reliably achieving pregnancy rates of 60–70% even with frozen–thawed semen (6, 10, 11). Beyond improving semen utilization efficiency, LAI has opened new opportunities for global germplasm exchange and conservation (3, 8). Nevertheless, most current studies have focused on isolated technical aspects, lacking a systems-level perspective that positions LAI at the core of reproductive management. With the rapid advancement of artificial intelligence, robotics, and the Internet of Things, LAI is now poised for transformative innovation (12, 13).

This review establishes a LAI-centered integrative framework to demonstrate how emerging technologies support critical decision points in LAI, while also assessing its economic feasibility and long-term prospects for the small ruminant industry.

## 2 Evolution and standardization of LAI technology

### 2.1 Historical development and technical maturation

The development of laparoscopic artificial insemination (LAI) exemplifies the convergence of veterinary surgery and reproductive biology. In 1982, Killeen and Caffery first reported successful intrauterine insemination in ewes using laparoscopy, marking the inception of this technique (9). Early LAI systems were bulky and technically demanding, requiring specialized surgical teams, which limited their adoption. During the 1990s, the systematic review published by Gourley and Riese signaled the maturation of the technique and its progression into commercial use (7). Their work detailed standardized procedures and identified critical success factors, laying the foundation for subsequent refinements. With advances in optical imaging, materials science, and anesthesiology in the early 21st century, LAI equipment became more portable and durable, while the surgical procedure itself became safer and faster (14, 15). Modern LAI is now highly standardized. Sathe’s procedural review provided comprehensive best practices, covering equipment selection, animal preparation, surgical techniques, and postoperative care (8). These refinements reduced procedure time to 5–10 min, substantially improving operational efficiency and animal welfare (8, 16) (Table 1).

**Table 1:** Historical milestones in the development of laparoscopic artificial insemination (LAI).

Year/Era	Key event	Major contributor(s)	Technical characteristics	Reference
1982	First report of LAI in sheep	Killeen and Caffery	Successful intrauterine insemination using laparoscopy	(9)
1990s	Standardization and commercialization	Gourley and Riese	Established standardized procedures and success criteria	(7)
Early 2000s	Modernization of equipment	Multiple researchers	Advances in imaging, materials, and anesthesia; portability	(14, 15)
2018	Procedural standardization review	Sathe	Detailed current best practices; reduced surgery time to 5–10 min	(8, 16)
2019	Technical recommendations	Gibbons et al.	Established global guidelines for LAI practice	(3)

### 2.2 Standardized procedures and key technical considerations

Successful LAI begins with meticulous planning and adherence to standardized protocols. The technical recommendations published by Gibbons et al. now serve as authoritative global guidelines for LAI practice (3). The key steps include:

#### 2.2.1 Animal selection and preparation

Selecting healthy ewes with a body condition score [Body condition score (BCS)] of 2.5–3.5/5 is essential for success. Animals should be free of reproductive disorders, aged 1–6 years, and in moderate body weight (3, 17). Preoperative evaluation typically includes general health checks, reproductive tract assessment, and basic blood biochemistry (17, 18).

#### 2.2.2 Estrus synchronization

Hormonal synchronization is critical for ensuring LAI success. The most common protocol involves vaginal sponges containing fluorogestone acetate [Fluorogestone acetate (FGA)] or medroxyprogesterone acetate [Medroxyprogesterone acetate (MAP)] for 12–14 days, followed by Pregnant mare serum gonadotropin (PMSG) injection at withdrawal to induce ovulation (19–22). Yu et al. compared five synchronization protocols and reported that Group II (11-day FGA + PGF2α on day 9 + 330 IU PMSG at withdrawal) yielded the best balance of lambing rate, twinning rate, and cost-effectiveness (19). Tirpan et al. further confirmed that 300 IU PMSG is sufficient for effective synchronization (20).

#### 2.2.3 Anesthesia and analgesia

Modern LAI typically combines local anesthesia with light sedation to minimize stress while ensuring smooth surgery (15, 17). Haan et al. compared CO₂ and medical air for abdominal insufflation, reporting no significant differences in respiratory parameters, though CO₂ was absorbed more quickly and improved postoperative recovery (16). Pain management has become integral to LAI, with preventive analgesia prior to surgery and extended postoperative pain control now considered best practice (15, 18, 23).

#### 2.2.4 Surgical technique

Feed is withheld for 12–24 h before surgery to reduce gastrointestinal distension. Following CO₂ insufflation, two 5–10 mm incisions are made for the laparoscope and surgical instruments. The uterine horns are located under direct visualization, and puncture is performed at well-vascularized, high-tension sites (8, 16). The choice of trocar system and insertion technique strongly influence outcomes; Kang et al. compared different trocar systems to guide optimal device selection (24).

#### 2.2.5 Semen handling and deposition

The thawing and preparation of frozen semen critically affect fertilization outcomes. Rickard and de Graaf outlined best practices, including optimal thawing temperature, extender choice, and sperm viability assessment (25). Typically, 0.1–0.2 ml of semen containing 20–50 × 10^6^ motile spermatozoa is deposited per uterine horn (25, 26).

### 2.3 Key factors influencing success rates

The success of laparoscopic artificial insemination (LAI) is shaped by multiple interacting factors. Recent large-scale studies have provided scientific evidence to quantify their relative importance. Hill et al., analyzing 28,447 commercial LAI cases, reported an overall pregnancy rate of 71.7% and identified key determinants, including progesterone source, PMSG dosage, semen type (fresh vs. frozen–thawed), breed, and operator workload (10). More recently, Spanner et al. developed a multivariate prediction model based on 30,254 ewes, refining the analysis of predictors with modern statistical methods (11) (Table 2).

**Table 2:** Major factors influencing LAI pregnancy outcomes.

Category	Specific factor	Level of influence	Optimal condition/recommendation	Reference
Ewe-related	Uterine tone	Most critical predictor	Pink, contractile upon touch, good vascularity	(11, 27)
	Age	Significant	Best outcomes at 2–4 years	(11, 27)
	Body condition score	Significant	BCS 2.5–3.5/5	(3, 17)
	Breed	Significant	Genetic background influences responsiveness	(31)
Semen-related	Motility	Determinant factor	Preservation during freezing–thawing process is critical	(26)
	Concentration	Key parameter	20–50 × 10^6^ motile sperm/0.1–0.2 ml	(25, 26)
Procedural	Timing of insemination	Crucial	52–56 h after sponge withdrawal	(28, 30)
	Deposition site	Important	Well-vascularized, high-tension region of the uterine horn	(8, 16)
Environmental	Season	Strong influence	Higher fertility during breeding season	(31)
	Nutritional status	Important	Adequate energy and protein supply	(31)

#### 2.3.1 Ewe-related factors

Uterine tone is the single most critical predictor, reflecting the receptivity of the uterine environment under hormonal regulation. A well-toned uterus is pink in color, contracts upon palpation, and shows strong vascularization (11, 27). Age, body condition score (BCS), breed, parity, and lactational status also significantly affect pregnancy outcomes. Young ewes (2–4 years old) generally have higher fertility, though primiparous animals may exhibit slightly lower rates due to heightened stress responses (11, 27).

#### 2.3.2 Semen-related factors

Sperm motility during the freezing–thawing process is a decisive determinant of fertility. Perkins et al. demonstrated that sperm motility and concentration directly affected pregnancy outcomes following LAI (26). Paulenz et al. further examined the role of extenders and storage conditions, providing technical support for the standardization of semen handling (28, 29).

#### 2.3.3 Procedural factors

The timing of insemination is critical. The optimal window is 52–56 h after sponge withdrawal, coinciding with follicular maturity just before ovulation (28, 30). O’Hara et al. showed that storage temperature and duration significantly affect the viability of fresh semen, informing guidelines for transport and short-term use (30). Interestingly, unilateral deposition yields result comparable to bilateral insemination but reduces surgical time and improves animal welfare (28, 30).

#### 2.3.4 Environmental and management factors

Seasonal effects, nutritional status, stress level, and overall flock management substantially influence pregnancy outcomes. Donovan et al. reported differences between natural and synchronized estrus conditions, while Fair et al. highlighted breed-specific responsiveness to LAI (31). Proper nutrition, stress reduction, and breeding during the natural season can significantly enhance reproductive success.

## 3 Integration of LAI with reproductive management

### 3.1 Optimization and innovation in Estrus synchronization

Estrus synchronization serves as the foundation for successful LAI, as its effectiveness directly determines insemination outcomes. Conventional protocols rely on combinations of progestogens and gonadotropins, yet new strategies continue to emerge as our understanding of small ruminant reproductive physiology deepens. Wildeus provided a seminal review outlining the theoretical framework of estrus synchronization in sheep and goats, emphasizing the pivotal role of PMSG in ovulation induction and the importance of tailoring dosages to breed, season, and individual variation (21). More recently, Habeeb highlighted the unique challenges of synchronizing seasonal breeders, further refining the theoretical basis for practical application (22). Field studies have confirmed these principles. Yu et al. compared five synchronization protocols in Hu sheep and demonstrated that Group II (11-day FGA + PGF2α on day 9 + 330 IU PMSG at withdrawal) achieved >80% estrus rate, the highest pregnancy outcomes, and up to 70% twinning rates, representing the most cost-effective solution for commercial farms (19). Tirpan et al. further showed that 300 IU PMSG was sufficient for synchronization, while higher doses increased the risk of excessive multiple births and dystocia (20). Earlier evidence by Mutiga et al. compared synchronization methods and PMSG dosages, providing historical support for dosage optimization (32). More recently, Shehabeldin et al. developed a PMSG-loaded chitosan nanoparticle system for controlled release, which enhanced synchronization efficiency and reduced the number of required injections, improving animal welfare (33). Alternative approaches have also been explored. Vallejo et al. compared Ovsynch with intravaginal progesterone devices in hair sheep and reported no significant differences in pregnancy rates, though Ovsynch was operationally simpler (34). Similarly, Deac et al. validated timed AI protocols during the non-breeding season, extending the applicability of LAI beyond seasonal constraints (35). In goats, Whitley and Jackson stressed that higher hormonal sensitivity requires species-specific dosage adjustments (36). A broader comparative review by Arya et al. summarized similarities and differences in synchronization strategies across cattle, sheep, and goats, offering insights for cross-species adaptation (37) (Table 3).

**Table 3:** Comparative outcomes of estrus synchronization protocols in small ruminants.

Protocol	Treatment scheme	Estrus rate (%)	Pregnancy rate (%)	Twinning rate (%)	Cost-effectiveness	Reference
Group I	Conventional progestogen-based protocol	–	–	–	Lower	(19)
Group II	11-day FGA + PGF2α on day 9 + 330 IU PMSG at removal	>80	Highest	~70	Most cost-effective	(19)
Group III	Alternative synchronization schemes	–	–	–	Moderate	(19)
300 IU PMSG	Standardized dose for induction	Effective	Stable	Moderate	Economical	(20)
Ovsynch protocol	Timed insemination, GnRH + PGF2α regimen	Comparable	No significant difference vs. progestogen	–	Easy to implement	(34)

### 3.2 Precision Estrus detection and optimization of insemination timing

Traditional estrus detection relies heavily on visual observation, which is labor-intensive, subjective, and prone to errors. With advances in computer vision and artificial intelligence, automated estrus detection has emerged as a practical solution. Yu et al. developed a multi-target detection neural network based on an improved YOLO v3 architecture, incorporating K-means++ clustering and additional detection layers, achieving 98.56% precision and 98.04% recall in identifying ewe mounting behavior (38). This provides an accurate “time anchor” for LAI. Later, Yu et al. introduced a lightweight network (EfficientNet-B0 with Senet modules), delivering 99.44% accuracy with a model size of only 40.6 MB and 48.39 FPS, suitable for real-time deployment (39). Complementary innovations include non-contact biometric monitoring. Fuentes et al. integrated visible and infrared thermal imaging with machine learning algorithms to assess heat stress in sheep (40). Such tools not only aid estrus detection but also expand to animal health monitoring. Additionally, Hu et al. applied an enhanced YOLOv5 model to identify grazing behaviors, while Yang et al. reviewed applications of computer vision for body condition scoring (41, 42). Integrating multiple sensing modalities—such as ultrasonography, temperature loggers, and accelerometers—offers a pathway toward comprehensive estrus monitoring. These multimodal decision-support systems are shifting insemination decisions from experience-based to data-driven, enabling a transition from group-level scheduling to precise individual-level timing (39, 40).

### 3.3 Integration with genetic improvement strategies

Laparoscopic artificial insemination (LAI) and genomic selection [Genomic selection (GS)] form a complementary pair of technologies that collectively accelerate genetic progress in small ruminants. Genomic selection, which leverages dense SNP chip data to estimate genomic breeding values [Genomic estimated breeding values (GEBVs)], provides unprecedented accuracy in identifying elite sires. LAI then acts as a “genetic multiplier,” enabling these elite sires to disseminate their germplasm broadly across ewe populations, unrestricted by geography or time (43, 44). Van der Werf and Banks emphasized the transformative potential of GS in sheep breeding, particularly its ability to shorten generation intervals and improve selection accuracy. Similarly, Daetwyler et al. demonstrated the value of whole-genome sequencing for dissecting complex traits, while Moghaddar et al. confirmed that GS outperforms pedigree-based approaches in young animals (43, 44). The economic implications of GS integration with LAI are substantial. Shumbusho et al. evaluated genomic selection in meat sheep breeding programs, showing that GS combined with reproductive technologies generates considerable returns on investment (45). In dairy sheep, Baloche et al. validated the accuracy of genomic predictions in the French Lacaune breed, providing technical assurance for genetic progress in milk traits (46).

An optimal integration strategy involves:

- Screening and selecting top young rams through GS.
- Collecting, freezing, and storing semen from these sires.
- Disseminating semen on a large scale via LAI.
- Feeding back progeny performance data to refine GS models.

This closed-loop “GS selection—LAI dissemination—data feedback” cycle maximizes genetic gain (45, 46).

Modern breeding programs must also align with sustainability goals. Brito et al. highlighted the importance of large-scale phenotyping to improve animal welfare and resilience (12). Likewise, González-Recio et al. incorporated methane emissions into dairy cattle breeding objectives, demonstrating the potential of genetic selection for environmental mitigation (13). Similar principles can be extended to small ruminants, where combining GS with LAI could yield breeds that are not only more productive but also climate-resilient and environmentally sustainable.

## 4 Emerging technologies empowering LAI

### 4.1 Artificial intelligence–driven prediction models and decision support

Artificial intelligence (AI) applications in LAI are transitioning from conceptual to practical. Spanner et al. developed a multivariate prediction model that integrates ewe characteristics, intraoperative uterine tone scores, and semen quality parameters to forecast pregnancy outcomes, achieving an AUC of 0.86 (11).

The model’s utility lies in several functions:

- Individualized selection—identifying ewes with extremely low predicted fertility, thus avoiding unnecessary costs and animal stress.
- Semen optimization—matching semen samples to ewes predicted to achieve the highest pregnancy rates, maximizing genetic resource efficiency.
- Dose adjustment—increasing insemination dose or applying bilateral insemination for borderline cases to improve outcomes.

Brito et al. emphasized that large-scale phenotyping is essential for training robust AI models with strong generalizability (12). More recently, Spanner et al. introduced a fertility model based on standardized *in vitro* semen thresholds, expanding AI-based predictive tools for field applications (29).

Ultimately, these AI systems are shifting LAI from an empirical practice to a data-driven, precision-guided intervention. The integration of genomic, environmental, and historical reproductive data will further enhance the personalization of reproductive management (12, 13) (Tables 4, 5).

**Table 4:** Comparative pregnancy outcomes of AI and conventional insemination methods in ruminants.

Insemination method	Semen type	Pregnancy rate (%)	Advantages	Limitations	Reference
LAI	Frozen–thawed	60–70	Bypasses cervical barrier; higher efficiency	Technically demanding; higher costs	(6, 10, 11)
LAI	Fresh	>70	Highest success rates	Short storage time for semen	(6, 10, 11)
TCAI	Fresh	40–60	Simple and low-cost	Anatomical limitations of cervix	(5, 6)
TCAI	Frozen–thawed	20–40	Low-cost	Poor efficiency; low success rates	(5, 6)
Cattle AI	Frozen–thawed	70–80	Well-established technique	Not directly applicable to small ruminants	(5, 6)

**Table 5:** Applications of emerging technologies in LAI and their outcomes.

Technology class	Specific innovation	Application function	Key outcomes/indicators	Development stage	Reference
Artificial intelligence	Multivariate prediction model	Pregnancy forecasting	AUC = 0.86	Field-tested	(11)
	Fertility model (*in vitro*)	Standardized threshold prediction	Improved predictive accuracy	Under development	(29)
Computer vision	YOLO v3–based detection	Estrus detection	Precision 98.56%, recall 98.04%	Field-tested	(38)
	Lightweight neural network	Real-time estrus monitoring	Accuracy 99.44%, FPS 48.39	Field-tested	(39)
	Non-contact biometrics	Heat stress monitoring	Visible + infrared imaging, ML modeling	Prototype	(40)
Robotics	Robotic-assisted LAI systems	Precision surgical assistance	Tremor filtration, motion scaling	Conceptual	(47–49)
	ZEUS robotic platform	Surgical assistance	Enhanced 3D visualization and fine motor control	Experimental	(50)
AR/VR	Augmented reality navigation	Intraoperative guidance	3D anatomical overlays	Prototype	(50–53)
	VR-based training	Skill acquisition	Safe and repeatable learning environment	Implemented	(18, 62)
IoT	Wearable sensors	Physiological monitoring	Body temperature, heart rate, activity patterns	Commercial use	(56)
	Digital twin systems	Individualized management	Virtual models for simulation and decision support	Conceptual	(58, 59)

### 4.2 Development and application of robotic-assisted surgery

Robotic-assisted surgery is already well established in human medicine and is gradually being adapted for veterinary use. These systems mitigate inherent limitations such as hand tremor and operator fatigue (47). For LAI, robotic systems offer several advantages:

- Enhanced flexibility and precision through robotic arms that exceed the range of human motion.
- Tremor filtration and motion scaling, which convert larger operator movements into precise micromovements (48).
- Improved 3D visualization, offering greater depth perception and clarity of surgical fields (47, 49).

Marescaux and Rubino described the ZEUS robotic platform in both experimental and clinical settings, highlighting its potential relevance for veterinary surgery (50). Although high costs remain a barrier, decreasing equipment prices and integration with AI and vision technologies could enable future “fully automated LAI workstations,” capable of completing insemination procedures from anesthesia to semen deposition with minimal human intervention.

### 4.3 Augmented reality and 3D imaging integration

Augmented reality (AR) overlays computer-generated information onto the real surgical field in real time, providing novel applications for LAI. Blixt and O’Brien systematically reviewed AR applications in veterinary surgery, reporting that AR can significantly enhance surgical accuracy and safety (51). In human medicine, Pratt et al. demonstrated that HoloLens-based AR systems can display three-dimensional (3D) vascular models intraoperatively, enabling surgeons to localize critical structures with greater precision (52). Similarly, Tepper et al. explored mixed reality integration, highlighting the combined potential of virtual and augmented reality in the operating room (53).

In LAI, AR applications could include:

- Intraoperative navigation: Overlaying virtual markers to guide precise puncture sites within the uterine horns.
- Critical structure annotation: Highlighting blood vessels and nerves to prevent iatrogenic injury.
- Remote guidance and training: Enabling experts to supervise procedures remotely in real time.

Advances in 3D imaging have provided a foundation for these AR applications. Fergo et al. demonstrated that 3D laparoscopy significantly improved depth perception and hand–eye coordination compared with 2D high-definition systems (54). Currò et al. further confirmed the value of 3D visualization for enhancing surgical precision (55). The combination of 3D/4 K laparoscopic imaging with AR guidance could transform LAI into a safer, more intuitive, and highly standardized procedure.

### 4.4 IoT and digital twin integration

The Internet of Things (IoT) leverages distributed sensor networks to provide continuous monitoring of animals and their environment. Neethirajan reviewed wearable biosensors that capture physiological parameters such as body temperature, heart rate, activity levels, and rumination behavior (56). Wolfert et al. emphasized IoT as a key enabler of smart farming, where data-driven insights underpin precision livestock management (57).

In the context of LAI, IoT systems can:

- Monitor ewe physiological indices (temperature, activity, feed intake) in real time using wearable devices.
- Track environmental parameters (temperature, humidity, air quality) with fixed sensors to ensure optimal housing conditions.
- Stream collected data to cloud-based platforms, providing AI-driven models with continuous updates (56, 57).

Digital twin technology represents the most advanced form of IoT integration. Rasheed et al. described digital twins as dynamic, data-driven virtual replicas that evolve alongside their physical counterparts (58). Pylianidis et al. further highlighted their agricultural potential, noting their ability to integrate multi-source data into predictive models (59).

For LAI, digital twins could create individualized ewe models that incorporate genomic profiles, reproductive histories, real-time physiological data, and environmental conditions. These virtual models can:

- Simulate different management strategies to predict reproductive outcomes.
- Continuously update based on real-time data, refining recommendations dynamically.
- Provide decision support to optimize insemination timing and protocols for each ewe.

This level of integration offers the potential for highly personalized reproductive management, transforming LAI from a population-level intervention into a truly individualized precision technology.

## 5 Economic evaluation and industrial challenges

### 5.1 Cost–benefit analysis and economic models

The economic evaluation of laparoscopic artificial insemination (LAI) requires a comprehensive assessment of direct costs, indirect benefits, and long-term impacts. Abbott conducted one of the earliest systematic cost–benefit studies on artificial insemination in wool sheep, concluding that LAI yields substantial returns on investment when the value of introduced germplasm is sufficiently high (60). Similarly, Valergakis et al. evaluated profitability in dairy sheep genetic improvement programs and found that LAI significantly enhanced milk yield and quality (61). More recent analyses by Myers et al. updated these models, incorporating the influence of modern technological advances on cost structures (6). Costs of LAI include equipment investment (laparoscopes, CO₂ insufflators, surgical tools), training and certification, semen procurement and storage, consumables, anesthesia and analgesics, and perioperative animal care (6, 60, 61). Benefits derive from higher pregnancy rates, improved semen utilization, rapid dissemination of elite genes, and reduced risks of sexually transmitted diseases compared with natural mating (6, 60, 61). Although single-procedure costs exceed those of transcervical insemination (TCAI), the superior pregnancy rates (60–70% vs. 20–40%) and long-term genetic and economic gains make LAI more profitable in structured breeding programs (6) (Table 6).

**Table 6:** Economic evaluations of LAI in sheep breeding programs.

Study/Author	Year	Study population	Main findings	Return on investment	Key factors	Reference
Abbott	1994	Wool-producing sheep	High ROI when genetic value is sufficient	Positive	Value of introduced genetics	(60)
Valergakis et al.	2010	Dairy sheep	Improved milk yield and quality	Significant	Enhanced productivity	(61)
Myers et al.	2025	Updated economic models	Technology advances lowered costs, improved outcomes	Improved	Modernization of tools	(6)
Hill et al.	1998	28,447 commercial ewes	Overall pregnancy rate 71.7% under field conditions	Favorable	Large-scale application	(10)

### 5.2 Major barriers to adoption and potential solutions

Despite its proven effectiveness, the widespread adoption of LAI is constrained by several challenges:

- High technical threshold: LAI requires specialized surgical skills and extensive practice, making operator training a major bottleneck. Solutions include standardized certification systems, competency-based evaluation, and training with VR/AR simulation tools. Mentorship programs can accelerate skill transfer across generations (18, 62).
- High equipment costs: The initial investment for LAI equipment remains a barrier for small-scale farms. Portable, integrated devices, leasing models, and government subsidies could lower adoption thresholds (63).
- Animal welfare concerns: LAI involves surgical procedures that require strict pain management protocols. Ensuring mandatory preoperative sedation, intraoperative monitoring, and postoperative analgesia is essential (15, 23).
- Lack of data standardization: Inconsistent data formats limit AI and digital system applications. Implementing FAIR principles (Findable, Accessible, Interoperable, Reusable) and establishing industry-wide data standards would facilitate large-scale integration (1) (Table 7).

**Table 7:** Major barriers to LAI adoption and proposed solutions.

Barrier category	Specific issue	Impact level	Proposed solution	Implementation difficulty	References
Technical	High skill requirements	High	Standardized training and certification	Moderate	(18, 62)
	Need for extensive practice	High	VR/AR-based surgical training	Moderate	(18, 62)
Economic	High equipment investment	High	Portable, integrated devices; leasing	High	(63)
	High entry cost for small farms	Moderate	Shared-use or subsidy programs	Moderate	(63)
Welfare	Surgical risk and pain	Moderate	Mandatory sedation and analgesia	Low	(23)
Data	Lack of interoperability	Moderate	FAIR-compliant open data platforms	High	(1)
	Absence of unified standards	Moderate	Industry-wide standardization	High	(1)

### 5.3 Industrial trends and policy support

The industrialization of LAI depends on synergistic advances in research, policy, and market forces. The *USDA Animal Genome Research Blueprint (2018–2027)* emphasized the importance of genomics and reproductive technologies in advancing livestock health, productivity, and welfare (2). Similar frameworks highlight the role of policy in supporting LAI’s development through research funding, workforce training, and international collaboration. On the market side, consumer demand for high-quality animal products, environmental sustainability pressures, and global competition are driving adoption. As Brito et al. observed for dairy cattle, future livestock systems must balance productivity with animal welfare and ecological sustainability (64). These principles equally apply to small ruminants, underscoring LAI’s relevance as both productivity-enhancing and sustainability-enabling technology.

## 6 Future perspectives and technological outlook

### 6.1 Technological convergence and system integration

Future developments in LAI will be characterized by high levels of integration across hardware and software. At the hardware level, the next generation of intelligent LAI workstations will likely incorporate high-definition 3D laparoscopes, robotic arms, AI decision modules, and automated semen-handling systems. These platforms could deliver fully automated workflows, from animal preparation to postoperative monitoring, reducing reliance on specialist operators (50).

On the software side, integrated platforms will unify multi-source data—IoT sensor readings, genomic profiles, historical reproductive records, and environmental metrics—into centralized decision-support systems. By leveraging advanced AI algorithms, these platforms will provide individualized reproductive recommendations, effectively building a comprehensive digital reproductive twin for each ewe (56–58).

### 6.2 Application of precision medicine principles

The adoption of precision medicine principles in veterinary science mirrors progress already achieved in human healthcare. In LAI, this will shift management from herd-level interventions to individualized protocols. Genomic data, obtained through whole-genome sequencing or SNP chips, will form the foundation of personalized reproductive planning by identifying genetic variants linked to fertility, disease resistance, or productivity traits (43, 44, 65–69). Epigenetic research will further expand this framework by uncovering how nutrition and environment regulate reproductive gene expression (70–76). Moreover, integrative multi-omics approaches—combining genomics, transcriptomics, proteomics, and metabolomics—will provide a systems-level view of reproductive physiology. These insights will enable targeted interventions that optimize insemination success while minimizing resource inputs (77–83).

### 6.3 Sustainability and environmental stewardship

The future of LAI must balance productivity gains with ecological sustainability. Thornton projected that livestock production will increasingly be judged by its environmental footprint (84), while Gerber et al. highlighted livestock’s central role in climate change mitigation (85).

LAI contributes to sustainability by:

- Improving reproductive efficiency, thereby reducing the resource demand of maintaining breeding flocks.
- Accelerating genetic progress, fostering animals that are more productive and resilient to climate stressors.
- Enabling precision management, which minimizes drug use, reduces greenhouse gas emissions, and lowers waste output.

Emerging methodologies such as life-cycle assessment [Life-cycle assessment (LCA)] will be essential for evaluating LAI’s environmental impact. Indicators like carbon footprint, water usage, and land-use efficiency will provide benchmarks to ensure that technological adoption aligns with sustainability goals (84, 85).

## 7 Conclusion and outlook

Over the past four decades, laparoscopic artificial insemination (LAI) has evolved from a novel surgical innovation to the gold standard of reproductive management in small ruminants. This review establishes a LAI-centered framework that highlights how emerging technologies—including AI, computer vision, robotics, IoT, and digital twins—are directly enhancing the precision, automation, and scalability of the technique.

Current LAI protocols already achieve stable pregnancy rates of 60–70% with frozen–thawed semen, a substantial improvement over transcervical methods. The integration of cutting-edge tools is driving LAI into a new era:

- AI models enable predictive, individualized reproductive management.
- Computer vision systems provide automated estrus detection with high accuracy.
- Robotics improve surgical precision and reduce operator variability.
- IoT and digital twins establish continuous, individualized reproductive monitoring.

Future development will likely focus on fully automated LAI workstations, multimodal data-driven decision systems, and non- or minimally invasive insemination technologies that further enhance efficiency while minimizing animal stress. Integration with genomic and multi-omics platforms will open new opportunities for climate-resilient, welfare-conscious breeding programs.

Ultimately, the trajectory of LAI reflects the broader transformation of livestock reproduction into a field defined by precision, sustainability, and ethical responsibility. With continued innovation and policy support, LAI has the potential to shape a resilient small ruminant industry capable of meeting the dual challenges of global food security and sustainable development.
