# Correction: Laparoscopic artificial insemination in small ruminants: technological integration, economic evaluation, and future perspectives

**DOI:** 10.3389/fvets.2026.1808846

**Published:** 2026-03-19

**Authors:** Ting-Chieh Kang, I-Ling Lai, Perng-Chih Shen

**Affiliations:** 1 Taiwan Livestock Research Institute, Ministry of Agriculture, Pingtung, Taiwan; 2 Graduate Institute of Bioresources, National Pingtung University of Science and Technology, Pingtung, Taiwan; 3 Department of Animal Science, National Pingtung University of Science and Technology, Neipu, Pingtung, Taiwan

**Keywords:** laparoscopic artificial insemination, small ruminants, precision reproductive management, artificial intelligence, computer vision, emerging technologies

In the published article, the reference for 1. Jacobsen A, de Miranda Azevedo R, Juty N, Batista D, Coles S, Cornet R, et al. FAIR principles in data management and stewardship. Encyclop Bioinform Comp Biol. (2017) 1:144–56 was incorrectly written.

It should be 1. Wilkinson MD, Dumontier M, Aalbersberg IJ, Appleton G, Axton M, Baak A, et al. The FAIR Guiding Principles for scientific data management and stewardship. *Sci Data*. (2016) 3:160018. doi: 10.1038/sdata.2016.18

In the published article the reference for 4. Abril-Parreño L, Druart X, Tsikis G, Cognié J, Pinczak A, Dubois A, et al. Cervical artificial insemination with frozen-thawed semen in sheep was incorrectly written.

It should be 4. Clínic de Barcelona. *New Technique Merges 3D and 4K Imaging for Gynaecological Surgeries*. Barcelona: Clínic de Barcelona (2021).

In the published article the reference for 14. Ceko MJ, Gebbie J. The application of 3D and 4K imaging in laparoscopy was incorrectly written.

It should be 14. Lee CL. The role of 3D laparoscopy in gynecology. *Taiwanese J Obstetr Gynecol*. (2025) 64:434–37. doi: 10.1016/j.tjog.2025.03.003

In the published article the reference for 15. Neves JP, Sargison ND. Pain management in sheep was incorrect.

It should be 15. Steagall PV, Bustamante H, Johnson CB, Turner PV. Pain management in farm animals: focus on cattle, sheep and pigs. *Animals*. (2021) 11:1483. doi: 10.3390/ani11061483

In the published article the reference for 17. Fisher A, Roadknight N. Welfare of sheep. CSIRO Publishing (2021) was incorrect.

It should be 17. Fisher AD, Roadknight N. Welfare of sheep. In: Abbott KA, editor. *Sheep Veterinary Practice*. Boca Raton, FL: CRC Press (2024).

In the published article the reference for 20. Tirpan G, Yilmaz O, Sönmez C. The effects of different doses of PMSG on estrus synchronization was incorrect.

It should be 20. Zonturlu AK, Özyurtlu N, Kaçar C. Effect of different doses of PMSG on estrus synchronization and fertility in Awassi ewes synchronized with progesterone during the transition period. *Kafkas Univ Vet Fak Derg*. (2011) 17:125–9. doi: 10.9775/kvfd.2010.2572

In the published article the reference for 22. Habeeb AA. Recent advances in estrus synchronization in small ruminants. (2021) was incorrect.

It should be 22. Cosentino IO, Balaro MFA, Menchaca A, Perez-Clariget R, Ungerfeld R, Brandão FZ. Recent advances in treatments for resynchronization of ovulation in small ruminants: a review. *Anim Repord*. (2023) 20:e20220111. doi: 10.1590/1984-3143-AR-2022-011

In the published article the reference for 42. Yang C, Zhai R, Wang H, Liu J, Zhao Y. Computer vision-based livestock body condition scoring was incorrect.

It should be 42. Yang SX, Han Y, Ma W, Tulpan D, Li J, Li J, et al. Review of computer vision for livestock body conformation assessment. *Aric Commun*. (2025) 3:100099. doi: 10.1016/j.agrcom.2025.100099

The original version of this article has been updated.

